# Covering and Re-Covering the Heart: Development and Regeneration of the Epicardium

**DOI:** 10.3390/jcdd6010003

**Published:** 2018-12-24

**Authors:** Yingxi Cao, Jingli Cao

**Affiliations:** Cardiovascular Research Institute, Department of Cell and Developmental Biology, Weill Cornell Medical College, New York, NY 10021, USA; caoyingxi@csu.edu.cn

**Keywords:** heart development, epicardium, proepicardial organ, heart regeneration, zebrafish

## Abstract

The epicardium, a mesothelial layer that envelops vertebrate hearts, has become a therapeutic target in cardiac repair strategies because of its vital role in heart development and cardiac injury response. Epicardial cells serve as a progenitor cell source and signaling center during both heart development and regeneration. The importance of the epicardium in cardiac repair strategies has been reemphasized by recent progress regarding its requirement for heart regeneration in zebrafish, and by the ability of patches with epicardial factors to restore cardiac function following myocardial infarction in mammals. The live surveillance of epicardial development and regeneration using zebrafish has provided new insights into this topic. In this review, we provide updated knowledge about epicardial development and regeneration.

## 1. Introduction

Heart disease is a leading cause of death worldwide. In the US alone, an estimated 900,000 people suffer a myocardial infarction (MI) each year [1], leading to cardiac muscle damage. Unfortunately, the adult mammalian heart has a limited ability to regenerate damaged cardiac muscle after heart attack [2]. Instead, the heart scars, leading to compromised function and a multitude of pathological sequelae. A therapeutic means to enhance the postinjury regenerative ability of the heart would have a tremendous clinical impact and has become one of the most challenging priorities for biomedical research. In the past decade, a number of studies have identified the epicardium, a mesothelial layer that envelops all vertebrate hearts, as a potential therapeutic target in heart repair. The epicardium is critical for heart development by serving as a progenitor pool and signaling center [3,4]. After heart injury, the epicardium serves as a source of paracrine signals for cardiomyocyte (CM) survival or division, as a supply of perivascular components and possibly other cell types such as CMs, and as a mediator of inflammation [5,6,7,8,9,10,11,12,13]. To include the epicardium in cardiac repair strategies, we need a comprehensive understanding of its role in heart development and regeneration. Here, we discuss the most recent progress and debates from the last few years, especially the new findings derived from live imaging in zebrafish embryos and explant culturing of adult zebrafish hearts.

## 2. The Proepicardial Organ

The cardiac epicardium was first observed more than a century ago by dissecting chick embryos, but was not confirmed until the late 1960s when electron microscopy was used [14,15,16]. In 1992 and 1993, Manner and colleagues found that the epicardial cells are derived from a transient embryonic cell cluster that forms at the venous pole of the developing heart tube, which they named pericardial villi [17,18]. Further studies in zebrafish, amphibians, rodents, and humans confirmed that the epicardial cells are derived from a similar transient cell cluster in the embryo now called the proepicardial (PE) organ (PEO). The PEO is a mesoderm-derived structure that gives rise to both the epicardium and coronary smooth muscle cells [19]. Although PEO formation and its cellular components have been studied for decades, a considerable amount of work is still needed to elucidate the cellular heterogeneity, progeny of PE cells and signals that guide PEO specification.

After PEO formation, the PE cells translocate and attach to the myocardial surface to form the epicardium. A few models of how PE cells translocate have been proposed. The first model suggests that a temporary bridge forms between the PE and myocardium and that PE cells migrate to the myocardial surface, as shown in the chick, *Xenopus* and axolotl models [20,21,22]. Cell translocation and attachment are regulated by BMP signaling in the atrioventricular canal [23]. A second model proposes that PE cells are released into the pericardial cavity as cell clusters (or cysts) and adhere to the myocardium around embryonic day 9.0 (E 9.0) in mice [24]. However, another study using mice suggested a third model, in which PE cells were transferred to the myocardium through both direct contacts between the PE and myocardium and adhesion of floating PE cell clusters to the myocardium [25]. Most recently, Li and coauthors found that both villous protrusions and floating cell clusters (or cysts) contribute to PE cell translocation to the myocardium in mice and that both processes rely on the cell division control protein CDC42 [26]. The authors confirmed the existence of physical contact between the PE and myocardium as an alternative mechanism and revealed that PE cells also migrate along the surface of the inflow tract to reach the ventricles [26]. Thus, PE cell translocation may involve multiple mechanisms concurrently. In humans, the PEO forms at Carnegie stage (CS) 11 (four weeks post conception), and the epicardium begins to cover the myocardial surface immediately afterwards [27,28,29]. Minimal evidence derived from examination of paraffin sections of human embryos suggested that villous protrusions of the PEO extending from the sinus wall contacted the ventricle on the dorsal side at CS 12 to facilitate epicardium formation [30]. Further studies are needed to dissect the details in human embryos.

**Figure 1:**
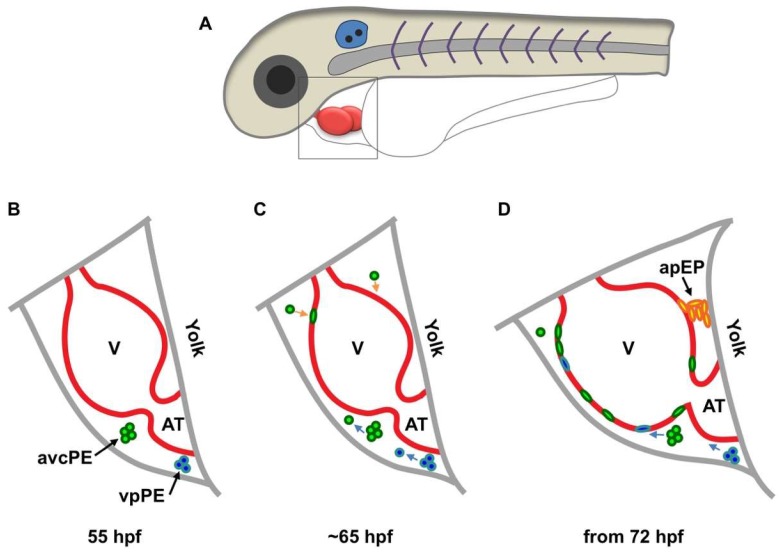
Schematic of epicardium formation in zebrafish. (**A**) Schematic of the anterior half of a zebrafish embryo. The framed region in (**A**) is enlarged to show details below (**B**–**D**); (**B**) at approximately 55 hpf, two PE clusters emerge from the mesothelial wall close to the atrioventricular canal (avcPE) and the venous pole PE (vpPE); (**C**) from approximately 65 hpf, cells are released (blue arrows) from the PE clusters and start to attach to the ventricular surface (orange arrows); (**D**) from 72 hpf, cells from the arterial pole epicardial precursor (apEP) pool (black arrow) are transferred to the ventricular surface through a cell bridge. V, ventricle; AT, atrium.

Two recent studies first described the mechanism in live animals using zebrafish embryos, which showed similar but partially contradictory findings. In one study, Peralta and colleagues performed live surveillance of PE formation and PE cell translocation to the myocardium using high-speed imaging and optical tweezing [31]. A significant portion of the epicardium and its precursor cells were labeled by enhanced green fluorescent protein (EGFP) under the control of the regulatory element of *Wilms tumor 1a* (*Wt1a*), a major epicardial transcription factor. This reporter labeled 70% of the PE cells. The authors defined three clusters of epicardial precursor cells: atrioventricular canal PE cells, venous pole (VP) PE cells, and arterial pole precursors (Figure 1). Cells from the first two PE clusters were released into the pericardial cavity and adhered to the ventricle between 60 and 72 h post fertilization (hpf, Figure 1). The pericardial fluid advections generated by the heartbeat are required for PE cluster formation, PE cell release and transfer to the myocardial surface, and the site of PE cell adhesion to the myocardium. When heart contractions were blocked, PE cluster formation and PE cell transfer were largely inhibited. The arterial pole precursors play only a minor role (contributing less than 10% of all epicardial cells) and do not derive from a PE cluster (Figure 1D). However, it does suggest the existence of direct contact between the precursor and the myocardium. Thus, epicardial precursor release and adhesion in zebrafish occur through both pericardial fluid advections and direct contact with the myocardium (Figure 1). It is worth mentioning that an avian study also identified the pericardial epithelium near the aortic sac as an origin of the distal outflow tract epicardium, which is morphologically and molecularly different from the PE-derived epicardium [32].

Another study in zebrafish performed by Plavicki and colleagues also supports the dual mechanism model [33]. PE and epicardial cells were traced using fluorescent reporters driven by the regulatory sequences of *transcription factor 21* (*tcf21*, a pan-epicardial marker in zebrafish [6]) and a cardiac enhancer trap line *pard3:GFP,* which is thought to mark the developing epicardium [34]. Reporter expression in fixed samples and live embryos illuminated that PE cells migrate through a cellular bridge formed between the pericardium and myocardium near the AV junction (Figure 1D). PE clusters near the VP and other smaller clusters on the pericardial wall were also observed close to the ventricle, with frequently seen free-floating aggregates in the pericardial space. In contrast to Peralta et al., blocking heart contractions does not interfere with PE emergence but impairs epicardial initiation. Further studies using heart explant cultures showed that inhibiting heart contractions did not prevent epicardial formation, implying that pericardial fluid is not required for epicardial formation. The difference between these two reports on heart contraction function might be due to the different markers and methods they used, although both reports were in agreement with the dual mechanism model. Of note, the reporter used by Peralta et al. only labeled 70% of PE cells. Thus, epicardial-specific markers are required for this field (see below). Additionally, how the in vitro assay would recapitulate in vivo situations is questionable. These and other studies in different species suggest that the mechanism might be species-specific [31,32,33], which needs further investigation. Although these two reports have some discrepancies caused by different markers and methods, both reports provide direct evidence of the dual mechanism model for epicardial formation.

## 3. Epicardial Cell Expansion and Differentiation

After transferring to the myocardial surface, the attached PE cells expand over the surface of the heart, including the ventricle, atrium and bulbous arteriosus (BA or outflow tract), to form a continuous cell layer. Cell polarity is essential for epicardial formation. In mice, mutation of PAR3, a key polarity protein, disrupts apical-basal polarity, and epicardial cells do not form cell cysts, although they migrate and proliferate [35]. The epicardium-specific Cdc42 deletion also disrupts cell polarity, decreases cell proliferation, and stops the formation of villous protrusions and floating epicardial clusters [26]. Cell adhesion proteins, such as EphrinB (cell surface ligand for the Eph tyrosine kinase receptor), VCAM-1 (vascular cell adhesion molecule 1), and integrins, are required for proper cell attachment and migration during epicardial formation [36,37,38,39]. Recently, Tran et al. found that the nuclear lamina protein lamin-B1 regulates epicardial cell migration through influencing the expression of cell adhesion genes, as *lamin-B1* null mice have delayed epicardial cell migration and reduced epicardial cell numbers [40].

Unlike other model systems, the human epicardium is multilayered. In humans, epicardial cells were first seen on the myocardial surface at CS11 [28]. Between CS14 and CS15, the entire human heart is covered by the epicardium. Interestingly, the human ventricular epicardium has multiple cell layers, while the atrial epicardium is a monolayer. The ventricular and atrial epicardial cells also have different morphologies and differentiation potentials [28]. In zebrafish, epicardial cells are first found on the ventricular surface at three days post fertilization (dpf), and the entire ventricle is covered at 4 to 5 dpf [41]. However, how epicardial cells expand to cover the entire heart is still unclear. For instance, what signals regulate epicardial cell proliferation and migration? Do these cells expand in a dispersed manner or as a continuous cell sheet? What are the cellular and molecular differences between atrial and ventricular epicardium?

After covering the heart tube, a subset of epicardial cells delaminate from the epicardium and undergo epithelial-to-mesenchymal transition (EMT), migrate into the subepicardium and give rise to epicardium-derived cells (EPDCs) [42]. Many signaling pathways have been reported to mediate epicardial cell EMT, such as retinoic acid (RA), fibroblast growth factor (FGF), Hippo/Yap, transforming growth factor beta (TGF-β), sonic hedgehog (SHH), Platelet-derived growth factor (PDGF), myocardin-related transcription factors (MRTFs), and extracellular matrix components (e.g., hyaluronic acid) [43,44,45,46,47,48,49,50,51,52,53,54]. For the cellular mechanism of epicardial EMT, Wu and colleagues found that the spindle orientation of epicardial cells controls cell entry into the myocardium [43]. In E12.5 and E13.5 mouse embryos, epicardial cells divide predominantly along two axes: either parallel to the basement membrane, keeping both daughter cells in the epicardium, or perpendicular to the basement membrane, resulting in one cell remaining in the epicardium while the other cell enters the myocardium. Targeted disruption of β-catenin in epicardial cells impaired adherens junctions and caused a randomized mitotic spindle orientation of epicardial cells, impairing the entrance of epicardial cells into the myocardium [43].

Epicardial cells are multipotent progenitors that can differentiate into multiple cardiac cell types during heart development, including cardiac fibroblasts, vascular smooth muscle cells (SMCs), atrioventricular (AV) cushion mesenchyme cells, endothelial cells and even CMs [19,55,56,57,58,59,60]. These progenies are often referred to collectively as EPDCs. However, only SMC and fibroblast fates are widely accepted, while the rest are largely controversial. A major reason for this controversy is the lack of epicardium-specific markers for genetic fate mapping, which could consistently label epicardial cells (active and quiescent) and their progenies. Widely used epicardial markers include *Tcf21*, *Wt1*, *T-Box 18* (*Tbx18*), *Scleraxis* (*Scx*), and *Semaphorin 3D* (*Sema3D*), each of which labels part of the epicardial population and/or labels additional cell types [58,59,61,62,63,64,65]. In addition, recent findings on the cellular heterogeneity of epicardial cells and EPDCs added complexity [66,67]. For instance, a single cell sequencing analysis of *tcf21^+^* epicardial cells in zebrafish revealed further heterogeneity within the epicardial population [67]. In this study, the authors performed transcriptome analysis on dozens of GFP^+^ cells purified from zebrafish carrying a *tcf21:nucEGFP* reporter. *tcf21*^+^ epicardial cells appear to include at least three subsets, defined by the expression signatures of potential candidate genes in each epicardial and EPDC subtype. These markers are useful tools for future lineage tracing experiments and functional studies of each subset in the epicardium, resolving the debate about epicardial lineage.

## 4. Epicardium in Heart Regeneration

Following cardiac injury in adult animals, the epicardial cells turn on embryonic genes (a process called activation) and proliferate, covering the injury site. Studies using zebrafish and mice have shown that the epicardium serves as a source of paracrine signals (including RA and neuregulin 1 (Nrg1)) and extracellular matrix (ECM) for CM survival or division [5,6,7,8,9,10,11,12,13]. It also serves as a supply of perivascular components (SMCs and pericytes) and other cell types (such as fibroblasts) and as a mediator of inflammation [5,6,7,8,9,10,11,12,13]. The fibroblasts ultimately contribute to cardiac fibrosis and scar formation. In contrast, the perivascular progeny is critical for coronary vessel growth. The epicardium mediates leukocyte recruitment and inflammatory responses after heart injury in mice, although it is unclear how that affects regeneration [5].

To directly test the impact of epicardium on heart regeneration, Wang and colleagues assessed epicardial injury responses in zebrafish by creating a transgenic line in which the bacterial nitroreductase (NTR) system driven by the regulatory sequence of *tcf21* (*tcf21:NTR*) ablates the epicardial cell population in adult fish after treatment with metronidazole [41]. Genetic depletion of epicardial cells after partial ventricular resection inhibited CM proliferation and delayed muscle regeneration, confirming the indispensable role of the epicardium in successful heart regeneration [41]. A recent intriguing study showed that a bioengineered collagen patch carrying the regeneration factor follistatin-like 1 (FSTL1) stimulated CM proliferation, reduced infarct size, and improved heart function after MI in mice and swine. Interestingly, only epicardial FSTL1 can promote regeneration [13]. Thus, understanding how the epicardium responds to cardiac injury and promotes heart repair will be the next priority for developing similar approaches to improve the inadequate regenerative capacity of human hearts after MI. Since zebrafish epicardial cells are heterogeneous [67], dissecting the contributions of each subtype to heart regeneration is essential. Precise manipulation of the correct population with proregenerative capacity would augment the regenerative response of the epicardium for therapeutic purposes. Moreover, a similar epicardial ablation tool using the diphtheria toxin fragment A (DTA) system has been developed in mice (*Wt1*^CreERT2^; *Rosa26*^DTA^), although the impact of epicardial ablation on heart regeneration has not been investigated [68]. It would be intriguing to test the requirement for epicardial cells in heart regeneration in neonatal mice, which can regenerate damaged hearts.

## 5. Regenerative Capacity of the Epicardium

**Figure 2:**
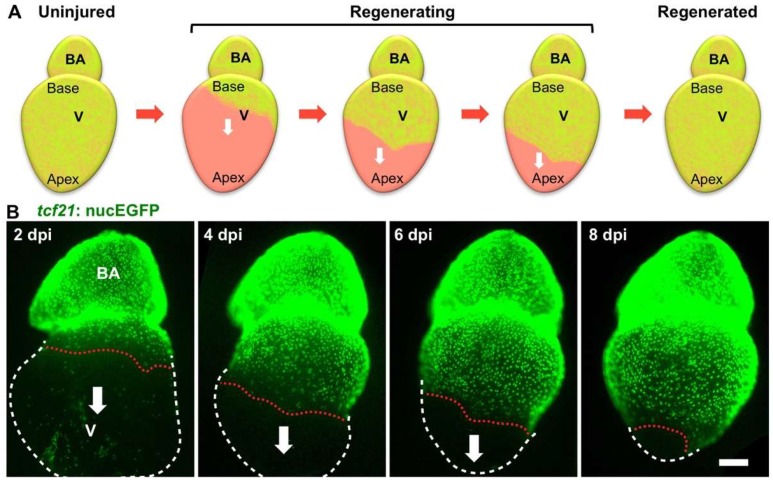
Epicardial regeneration. (**A**) Schematic for epicardial ablation and regeneration. The epicardium is shown in green color. The white arrows indicate the directions of epicardial cell migration; (**B**) explanted adult zebrafish hearts were imaged daily post epicardial ablation. The epicardial cell sheet regenerates along the ventricular surface in a base-to-apex direction (arrows). The nuclei of epicardial cells are labeled with nuclear GFP (*tcf21:*nucEGFP). White dashed lines outline the ventricles, and red dashed lines indicate the leading edge of the migrating epicardial cell sheet. Scale bar: 200 μm. BA, bulbous arteriosus; v, ventricle; dpi, days post injury.

A damaged zebrafish heart can restore the lost epicardium, suggesting a regenerative capacity of the epicardium. To assess epicardial cell regeneration, Wang and colleagues analyzed the cellular behavior of spared epicardial cells after a massive loss of epicardial cells in adult zebrafish [41]. Real-time imaging of epicardial cells in explanted hearts demonstrated that the epicardium possesses a remarkable regenerative ability to restore the lost cell mass in approximately two weeks, even after losing 90% of its cells. Upon ablation, the spared epicardial cells proliferate and migrate as a sheet to cover the exposed ventricular surface in a wave from the ventricular base toward its apex (Figure 2) [41]. The spared epicardial cells without contact with the ventricular base or the migrating cell sheet do not proliferate or migrate until they converge with migrating cell sheets from the ventricular base. This observation suggests potential signals from the ventricular base, which has direct contact with the BA. Interestingly, the study in explanted hearts showed that the BA is essential for epicardial regeneration and regulates the direction of epicardial cell migration. Further mechanistic studies suggested that the Hedgehog (Hh) ligand expressed in the BA drives epicardial regeneration. Which cells in the BAs secrete Hh and how the signal exerts local effects are still elusive. Potentially, a signaling gradient (together with other factors) is established along the base-apex axis to direct regeneration. In addition, the embryonic epicardial cells also migrated from the ventricular base toward the apex after genetic ablation at 3 dpf [41]. Whether this regeneration alongside development is regulated in the same way is unclear. Moreover, whether the developing epicardium is expanded on the heart surface in a similar manner is also intriguing (Figure 1). In addition, the epicardial ablation tool in mice (*Wt1*^CreERT2^; *Rosa26*^DTA^) might be useful for testing the regenerative capacity of the epicardium in mammals [68]. Collectively, zebrafish are an ideal model for live surveillance of epicardial cell development and regeneration, providing new insights into epicardial biology and inspiring new studies in mammalian hearts.

**Figure 3:**
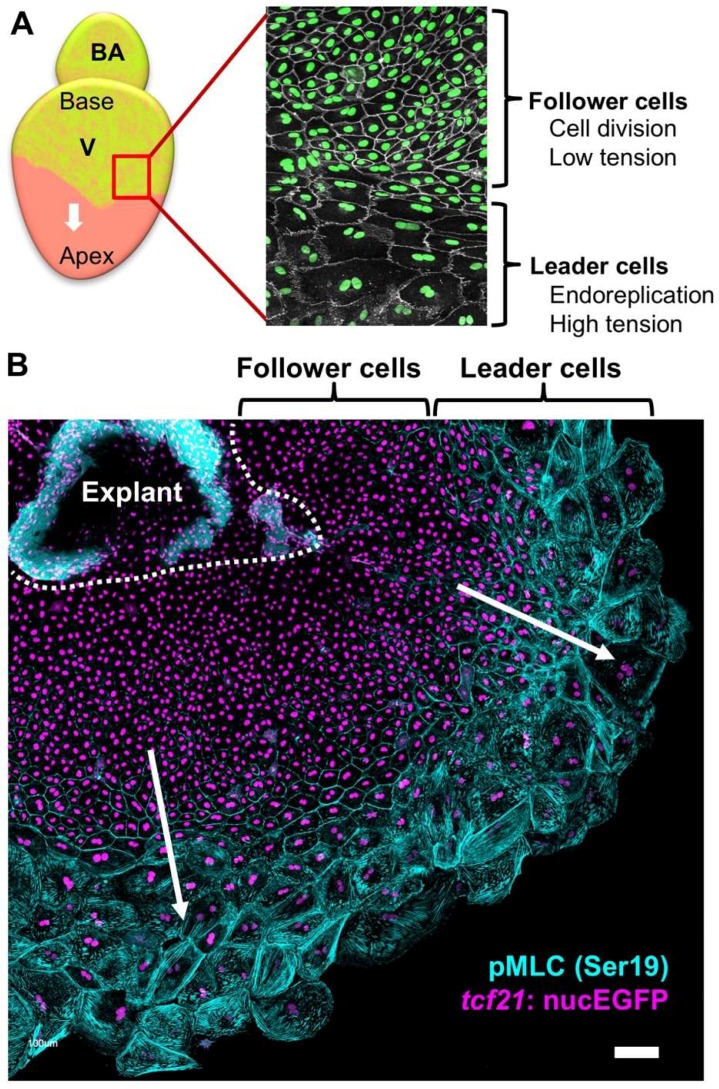
Two regions of the regenerating epicardial cell sheet. (**A**) Schematic of epicardial regeneration. The detail of the framed region of the regenerating epicardium is shown on the right. Nuclei are shown in green while cells are outlined in white. The epicardium forms two distinct domains: a leading edge of large, multinucleated leader cells followed by small, mononucleated follower cells. The follower cells have low cellular tension and undergo cell division, while the leader cells have high cellular tension and undergo endoreplication. (**B**) Migration of an epicardial cell sheet cultured in vitro showing two regions. Epicardial nuclei are labeled with *tcf21:*nucEGFP in violet. Immunostaining against phosphorylated myosin light chain 2 (pMLC, at Ser19, in cyan) was used as an indicator of mechanical tension. Scale bar: 100 μm.

To further dissect the cellular and molecular details of epicardial regeneration, in a follow-up study, the authors found that epicardial cell regeneration involves spatiotemporally guided, transient endoreplication (increasing cell ploidy), in addition to regular cell division (Figure 3) [69]. Endoreplication is an emerging theme in regenerative biology, in which cells replicate DNA once or more but do not undergo cytokinesis [70,71,72]. The authors observed large, multinucleated cells on the ventricular surface during epicardial regeneration – both in vivo and ex vivo. This phenomenon was restricted to the cells at the leading edge of the migrating epicardial sheet (leader cells), while the trailing cells (follower cells) possessed small cell size and nuclei, comparable to uninjured ones (Figure 3). Endoreplication includes endocycling, in which cells synthesize DNA without cell or nuclear division, and endomitosis, in which cells divide without cytokinesis [72]. With FUCCI (Fluorescent Ubiquitination-based Cell Cycle Indicator) reporters to visualize the cell cycle phases [73], the authors were able to distinguish endocycling and endomitosis from normal cell division. They found that both endocycling and endomitosis, but not cell fusion at the leading edge of migrating cell sheets, led to polyploidy of the leader cells. A further mechanistic study using explant culture suggested that higher mechanical tension at the leading edge than in the follower cells is the cause of polyploidy (Figure 3) and that elevated mechanical tension by physical stretch leads to extra polyploidy, even in the follower cells. When the ventricular surface was fully repopulated at approximately 14 days postinjury (dpi), only small cells were observed, suggesting transient endoreplication during epicardial regeneration. Live imaging analysis of heart explants indicated that nearly 80% of polyploid nuclei ended with nuclear fragmentation, indicating that the polyploid cells are eventually eliminated through apoptosis. Further pharmacological studies showed that when epicardial cell proliferation is inhibited, regeneration occurs from an extremely limited number of large cells that undergo endoreplication. In contrast, augmenting epicardial cell proliferation largely decreased the incidence of polyploidy. In addition, upon laser ablation of the leader cells in explant culture, the follower cells that faced the migration front underwent endoreplication to reconstruct the leader region. The authors concluded that the polyploid leader cells benefit the regenerative process by covering the ventricular surface more efficiently than normal diploid cells. However, unsolved questions remain: what constitutes the tissue tension in vivo; whether polyploidy is essential for epicardial regeneration as it is in epidermal regeneration in fly [70]; what is the difference between endocycling and endomitosis; and how the polyploid cells are eventually eliminated after regeneration. This study reveals new gaps in our understanding of tissue regeneration that will lead to years of exciting explorations.

## 6. Conclusions and Perspectives

Studies of epicardial regeneration have brought several conceptual advances that affect how we think of tissue regeneration. First, tissue regeneration can be achieved through either cell division or endoreplication, suggesting plasticity in regeneration approaches. Polyploidization is required for wound healing in the adult *Drosophila* epithelium to stabilize wounds mechanically, and inhibition of polyploidization impairs wound closure [70]. Conversely, the polyploidy of CMs seems to be a barrier to heart regeneration in both mammals and zebrafish [74,75]. The benefits of polyploidy might be tissue-specific. Second, polyploidy can be eliminated through apoptosis upon completion of regeneration. By contrast, the polyploid syncytium during wound healing in the adult *Drosophila* is permanent once formed [70]. It is unclear what differentiates these processes and what triggers apoptosis. Comparative studies on this tissue specificity may be informative for manipulation of polyploidy for heart muscle regeneration (e.g., switching from endoreplication to cell division). Third, mechanical tension is a key regulator of cell cycle decision (division vs. endoreplication), and altering tissue tension can be utilized to improve tissue regeneration.

Tissue regeneration is often achieved by reactivating the developmental program, such as heart regeneration [76,77]. The finding of epicardial regeneration implies unexplored features of epicardial development. Further exploration of how epicardial cell expansion on the heart surface is regulated, particularly whether polyploidy and directional cell migration are involved, would be intriguing. The ultimate goal of studying the epicardium is to develop the epicardium as a therapeutic target for heart repair in humans. To achieve this, we first need to define the subsets of epicardial cells and identify specific markers for epicardium-derived lineages. These markers enable definition of the signals regulating the epicardial fate and precise manipulation of the epicardium and its lineages for regeneration. Second, developing new techniques and methods that support both mechanistic and large-scale approaches will advance epicardial biology and promote endeavors aimed at heart repair. Finally, understanding the mechanisms by which the epicardium contributes to innate heart regeneration in lower model systems such as zebrafish will continue to build a basis for therapeutic strategies of heart repair.
